# SARS-CoV-2 in Quarantined Domestic Cats from COVID-19 Households or Close Contacts, Hong Kong, China

**DOI:** 10.3201/eid2612.202786

**Published:** 2020-12

**Authors:** Vanessa R. Barrs, Malik Peiris, Karina W.S. Tam, Pierra Y.T. Law, Christopher J. Brackman, Esther M.W. To, Veronica Y.T. Yu, Daniel K.W. Chu, Ranawaka A.P.M. Perera, Thomas H.C. Sit

**Affiliations:** City University of Hong Kong, Hong Kong, China (V.R. Barrs);; The University of Hong Kong, Hong Kong (M. Peiris, D.K.W. Chu, R.A.P.M. Perera);; Government of the Hong Kong Special Administrative Region, Hong Kong (K.W.S. Tam, P.Y.T. Law, C.J. Brackman, E.M.W. To, V.Y.T. Yu, T.H.C. Sit)

**Keywords:** severe acute respiratory syndrome coronavirus 2, SARS-CoV-2, coronavirus disease, COVID-19, coronavirus, viruses, pandemic, quarantine, domestic cats, households, close contacts, respiratory infections, zoonoses, Hong Kong, China

## Abstract

We tested 50 cats from coronavirus disease households or close contacts in Hong Kong, China, for severe acute respiratory syndrome coronavirus 2 RNA in respiratory and fecal samples. We found 6 cases of apparent human-to-feline transmission involving healthy cats. Virus genomes sequenced from 1 cat and its owner were identical.

Naturally occurring human-to-animal transmission of severe acute respiratory syndrome (SARS) coronavirus was reported during 2003 when viral RNA was detected in oropharyngeal and rectal swab specimens from healthy domestic cats in a housing estate at the center of a large SARS cluster in Hong Kong, China; infections were confirmed serologically (*1*). Susceptibility of cats to infection with this virus and transmission between cats were demonstrated experimentally (*2*). Pulmonary pathologic changes, similar to those for humans with SARS, developed in infected cats, but the cats remained asymptomatic (*2*,*3*).

These findings informed the current precautionary strategy of the Agriculture, Fisheries and Conservation Department of Hong Kong to quarantine mammalian pets from households with confirmed human coronavirus disease (COVID-19) or their close contacts (defined as a person who had face-to-face contact for >15 min with a person who had confirmed SARS coronavirus 2 [SARS-CoV-2] infection) in a holding facility, when alternative care was unavailable. Pets are swabbed for SARS-CoV-2 testing and confined until reverse transcription PCR (RT-PCR) results are negative on 2 consecutive occasions. Findings for 2 infected dogs have been reported (*4*). We report testing results for cats.

Swab (nasal, oral, rectal) specimens and feces collected from nonsedated cats after admission were tested for SARS-CoV-2 RNA at the Agriculture, Fisheries and Conservation Department Veterinary Laboratory by using a commercial RT-PCR targeting the partial envelope and RNA-dependent RNA polymerase genes (Molbiol Lightmix; TIB MOLBIOL, https://www.tib-molbiol.com). This PCR does not show cross-reactivity with these genes from enteric coronavirus (*5*).

For samples with positive or equivocal results, confirmatory quantitative RT-PCRs targeting nonstructural protein 4, nonstructural protein 16, nucleoprotein, and membrane genes were performed at the World Health Organization COVID-19 Reference Laboratory at the University of Hong Kong (*4*). Animals with positive results were evaluated by repeated sampling to monitor viral shedding by RT-PCR. Serologic analysis was used selectively.

We sampled 50 cats during February 11–August 11, 2020. Time from onset of COVID-19 symptoms in owners to first sampling of their cats was available for 21 owners of 35 cats and ranged from 3 to 15 (median 8, interquartile range 4) days. SARS-CoV-2 RNA was detected in samples from 6 (12%) of 50 cats (Table, https://wwwnc.cdc.gov/EID/article/26/12/20-2786-T1.htm).

The first positive case (cat 1) was from a household that had 3 persons with confirmed cases of COVID-19; their symptoms (fever, cough, or shortness of breath) started on March 20, 29, and 30, 2020. Their 7-year old, female, domestic shorthair cat was examined by a veterinarian at admission on day 1 (March 30), and reported to be clinically healthy. Nasal, oral, and rectal swab specimens collected on day 1 were positive for SARS-CoV-2 RNA; viral nucleoprotein gene copy number were log_10_ 6.3/mL, log_10_ 5.6/mL, and 3.2 log_10_/mL, respectively.

Attempts to culture virus from day 1 samples on Vero E6 (ATCC CRL-1586) cells as described (*4*) were unsuccessful. Viral RNA was detected in oral swab specimens for 8 days and nasal swab specimens for 11 days, but rectal swab specimens were negative after day 1 (Table).

**Table:** Results of reverse transcription PCR for detection of severe acute respiratory syndrome coronavirus 2 in nasal, oral, rectal, and fecal swab specimens for 6 cats, Hong Kong, China, 2020*

Animal breed/age, y/sex	Date tested	E gene, cutoff <36					RdRP gene, cutoff <40					NSP14 gene, cutoff <40					NP gene, cutoff <40					NSP16 gene, cutoff <40					M gene			
		N	O	R	Fe		N	O	R	Fe		N	O	R	Fe		N	O	R	Fe		N	O	R	Fe		N	O	R	Fe
DSH/7/F	Mar 30	**21.3**	**22.9**	**33.0**	ND		**32.0**	**32.0**	**38.9**	ND		**26.8**	**29.6**	**38.0**	ND		**26.4**	**29.3**	**38.0**	ND		**27.4**	**32.3**	**38.9**	ND		**26.0**	**28.8**	**36.9**	ND
	Apr 1	**35.5**	**26.7**	Neg	ND		**Neg**	**35.5**	Neg	ND		Neg	**30.6**	Neg	ND		Neg	**31.7**	Neg	ND		Neg	**30.5**	Neg	ND		**38.6**	**29.6**	Neg	ND
	Apr 3	**37.3**	**37.1**	Neg	ND		Neg	Neg	Neg	ND		Neg	Neg	Neg	ND		**39.9**	Neg	Neg	ND		Neg	Neg	Neg	ND		Neg	Neg	Neg	ND
	Apr 6	Neg	**35.9**	Neg	Neg		Neg	Neg	Neg	Neg		ND	Neg	ND	ND		ND	Neg	ND	ND		ND	Neg	ND	ND		ND	Neg	ND	ND
	Apr 9	**38.6**	Neg	Neg	Neg		Neg	Neg	Neg	Neg		**38.4**	ND	ND	ND		Neg	ND	ND	ND		Neg	ND	ND	ND		Neg	ND	ND	ND
	Apr 14	Neg	Neg	Neg	Neg		Neg	Neg	Neg	Neg		Neg	Neg	Neg	Neg		Neg	Neg	Neg	Neg		Neg	Neg	Neg	Neg		Neg	Neg	Neg	Neg
	Apr 15	Neg	Neg	Neg	ND		Neg	Neg	Neg	ND		Neg	Neg	Neg	ND		Neg	Neg	Neg	ND		Neg	Neg	Neg	ND		Neg	Neg	Neg	ND
DSH/3/M	Jul 13	Neg	Neg	Neg	**37.2**		Neg	Neg	Neg	Neg		ND	ND	ND	Neg		ND	ND	ND	**37.2**		ND	ND	ND	**40.0**		ND	ND	ND	**39.0**
	Jul 14	Neg	Neg	Neg	ND		Neg	Neg	Neg	ND		ND	ND	ND	ND		ND	ND	ND	ND		ND	ND	ND	ND		ND	ND	ND	ND
	Jul 15	Neg	**37.3**	Neg	**36.2**		Neg	Neg	Neg	Neg		ND	Neg	ND	Neg		ND	Neg	ND	Neg		ND	Neg	ND	Neg		ND	Neg	ND	Neg
	Jul 17	ND	ND	ND	ND		ND	ND	ND	ND		ND	ND	ND	ND		ND	ND	ND	ND		ND	ND	ND	ND		ND	ND	ND	ND
	Jul 20	Neg	Neg	Neg	ND		Neg	Neg	Neg	ND		ND	ND	ND	ND		ND	ND	ND	ND		ND	ND	ND	ND		ND	ND	ND	ND
	Jul 21	ND	ND	ND	Neg		ND	ND	ND	Neg		ND	ND	ND	ND		ND	ND	ND	ND		ND	ND	ND	ND		ND	ND	ND	ND
	Jul 23	Neg	Neg	Neg	ND		Neg	Neg	Neg	ND		ND	ND	ND	ND		ND	ND	ND	ND		ND	ND	ND	ND		ND	ND	ND	ND
	Jul 24	ND	ND	ND	Neg		ND	ND	ND	ND		ND	ND	ND	ND		ND	ND	ND	ND		ND	ND	ND	ND		ND	ND	ND	ND
ASH/13/M	Jul 21	Neg	**22.0**	Neg	ND		Neg	**27.3**	Neg	ND		Neg	**31.0**	Neg	ND		Neg	**30.7**	Neg	ND		ND	**31.6**	ND	ND		ND	**30.3**	ND	ND
	Jul 24	Neg	**29.0**	Neg	ND		Neg	**34.9**	Neg	ND		ND	**34.7**	ND	ND		ND	**34.9**	ND	ND		ND	ND	ND	ND		ND	ND	ND	ND
	Jul 27	ND	ND	ND	Neg		ND	ND	ND	ND		ND	ND	ND	ND		ND	ND	ND	ND		ND	ND	ND	ND		ND	ND	ND	ND
	Jul 30	Neg	Neg	Neg	Neg		Neg	Neg	Neg	Neg		ND	ND	ND	ND		ND	ND	ND	ND		ND	ND	ND	ND		ND	ND	ND	ND
	Aug 4	Neg	Neg	Neg	ND		Neg	Neg	Neg	ND		ND	ND	ND	ND		ND	ND	ND	ND		ND	ND	ND	ND		ND	ND	ND	ND
DSH/2/F	Jul 28	Neg	Neg	**34.2**	**32.7**		Neg	Neg	**39.5**	**34.5**		ND	ND	**36.4**	**35.5**		ND	ND	**37.5**	**36.9**		ND	ND	ND	ND		ND	ND	ND	ND
	Aug 4	Neg	Neg	Neg	Neg		Neg	Neg	Neg	Neg		ND	ND	ND	ND		ND	ND	ND	ND		ND	ND	ND	ND		ND	ND	ND	ND
	Aug 7	Neg	Neg	Neg	Neg		Neg	Neg	Neg	Neg		ND	ND	ND	ND		ND	ND	ND	ND		ND	ND	ND	ND		ND	ND	ND	ND
SSH/5/M	Jul 31	Neg	**26.8**	Neg	Neg		Neg	**33.1**	Neg	Neg		Neg	**32.7**	Neg	ND		Neg	**29.7**	Neg	ND		ND	ND	ND	ND		ND	ND	ND	ND
	Aug 4	**34.6**	**35.3**	**36.5**	**32.7**		**40.0**	Neg	Neg	**37.4**		Neg	Neg	Neg	**37.0**		**37.3**	**37.9**	Neg	**34.1**		ND	ND	ND	ND		ND	ND	ND	ND
	Aug 11	Neg	Neg	Neg	Neg		Neg	Neg	Neg	Neg		ND	ND	ND	ND		ND	ND	ND	ND		ND	ND	ND	ND		ND	ND	ND	ND
DSH/5/F	Aug 4	Neg	**36.0**	Neg	ND		ND	**40.0**	ND	ND		ND	**37.2**	ND	ND		ND	Neg	ND	ND		ND	ND	ND	ND		ND	ND	ND	ND
	Aug 11	Neg	Neg	Neg	Neg		Neg	Neg	Neg	Neg		ND	ND	ND	ND		ND	ND	ND	ND		ND	ND	ND	ND		ND	ND	ND	ND
	Aug 13	Neg	Neg	Neg	ND		Neg	Neg	Neg	ND		ND	ND	ND	ND		ND	ND	ND	ND		ND	ND	ND	ND		ND	ND	ND	ND

*Values are cycle thresholds. Values in bold are positive or equivocal results greater than the cutoff value. ASH, American shorthair; DSH, domestic shorthair; E, envelope; Fe, fecal; N, nasal; Neg negative; ND, not determined; NP, nucleoprotein; NSP, nonstructural protein; O, oral; R, rectal; rectal; RdRp, RNA-dependent RNA polymerase; SSH, Scottish shorthair.

We performed serologic analysis to detect neutralizing antibodies by using a 90% plaque reduction neutralization test for SARS-CoV-2 (*4*). The result was positive for the only serum sample collected (on day 19) and had titer >1:320 (*4*).

Viral genomes from cat 1 and 1 owner were sequenced by using a MiSeq Sequencing Platform (Illumina, https://www.illumina.com) after reverse transcription of viral RNA and multiple, overlapping, »2-kb PCRs that targeted the viral genome (*4*). Both genome sequences (29,830 nt sequenced; 99.8% of the genome) were identical (Figure) and deposited in GenBank (accession no. MT628701).

**Figure:**
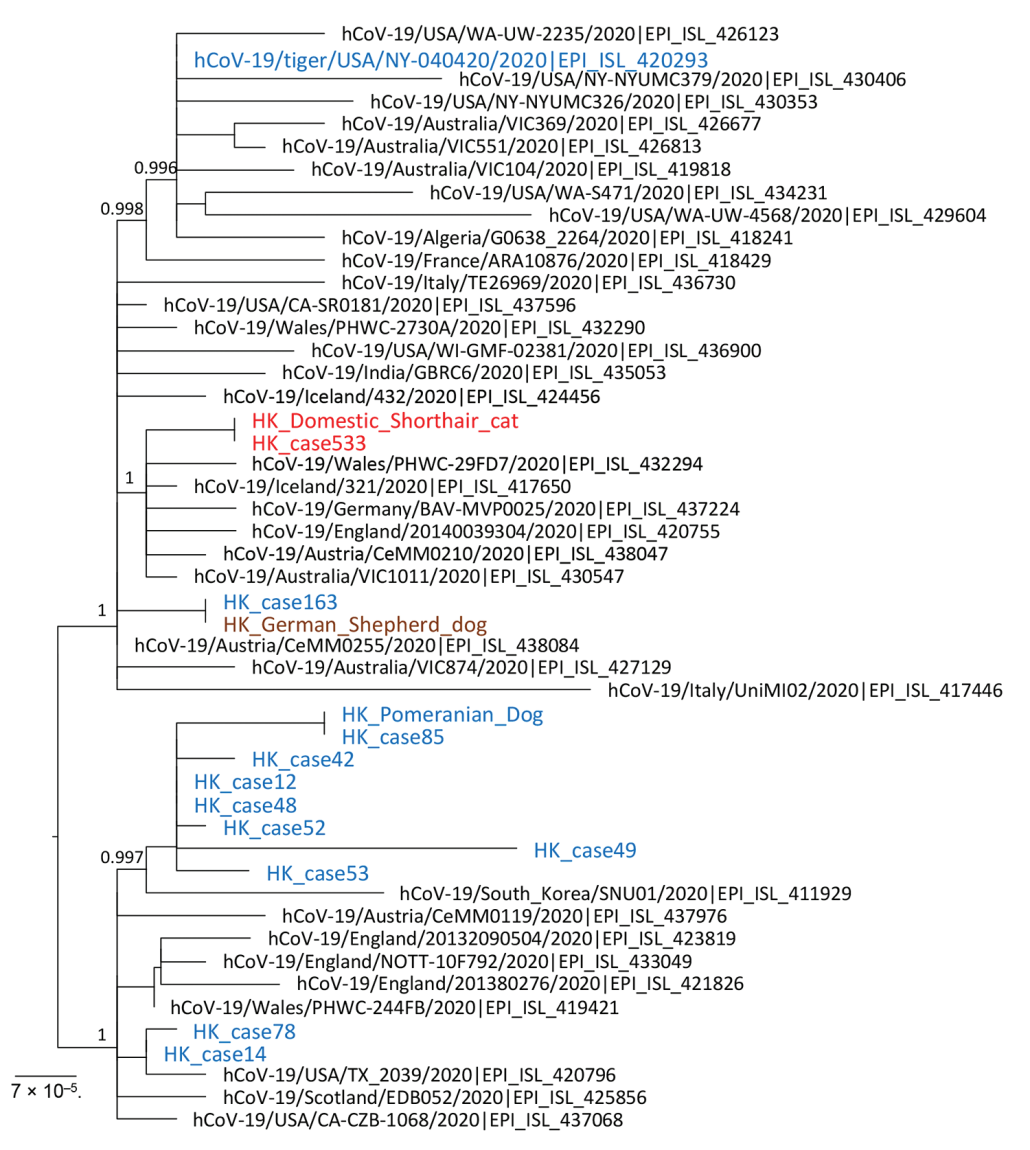
Phylogenetic analysis of SARS-CoV-2 full genome from an infected cat and the human index case-patient, Hong Kong, China. A virus sequenced directly from a tiger in a zoo in United States was included in this analysis. Virus genome alignment was prepared and manually trimmed at genome 5¢ and 3¢ ends for low-alignment quality. A resulting alignment of 29,655 nt was analyzed by using PhyML (http://www.atgc-montpellier.fr) and the generalized time reversible nucleotide substitution model. Branch support identified by using the fast approximate likelihood ratio test are shown at major nodes. The Hong Kong feline virus from cat 1 and that of its owner are shown in red. Canine and human viruses from Hong Kong, including from the dogs’ owners (HK_case 163 and HK_case 85) are shown in blue. Numbers along branches are bootstrap values. Scale bar indicates nucleotide substitutions per site. SARS-CoV-2, severe acute respiratory syndrome coronavirus 2.

Four of the other 5 positive cats were from confirmed COVID-19–infected households, and 1 indoor-only cat belonged to a close contact who was not confirmed to be infected. Time from onset of COVID-19 symptoms in owners to animal sampling was known for 3 cats (5, 11, and 8 days); 1 had equivocal envelope gene PCR results but was positive by a novel surrogate virus neutralization test (*6*). Signs of disease did not develop in any infected cats, consistent with experimental feline infections, which are also usually subclinical (*7*; A. Bosco-Lauth et al., unpub. data).

COVID-19–like signs have been reported in domestic cats naturally infected with SARS-CoV-2 in other countries (*8*). In addition, 4 tigers and 3 lions with respiratory signs in a zoo in New York, New York, USA, were confirmed to be shedding SARS-CoV-2 in feces (S.L. Bartlett et al., unpub. data). Susceptibility to SARS-CoV-2 might differ between felid species.

SARS-CoV-2 RNA persisted longest in nasal secretions in 1 case for 11 days at low levels. Viral RNA was detected in nasal washes from kittens experimentally inoculated with SARS-CoV-2 for 8–9 days, after which sampling was stopped (*7*). Cats acquiring infection from being cohoused with experimentally infected cats shed virus in respiratory secretions longer (7 days) than directly inoculated cats (5 days) (*8*).

Although feline-to-human transmission is theoretically possible, we did not find any evidence of this transmission. The timeline of infection in cat 1 and the finding of an identical SARS-CoV-2 genome sequence in a human from the same household is consistent with human-to-animal transmission. In support of these findings, the cat had no outdoor access.

More widespread serologic surveillance of cats in contact with COVID-19 patients is warranted to determine the prevalence of human-to-cat transmission. Some infected cats might have stopped shedding virus before being quarantined because viral shedding periods as short as 3 days have been reported in experimentally infected cats (*7*).
